# Two Contrasting Scintigraphy‐Negative Pediatric Meckel's Diverticula Identified and Localized by Intestinal Ultrasound and Double‐Balloon Enteroscopy: A Report of Two Cases

**DOI:** 10.1002/deo2.70379

**Published:** 2026-07-13

**Authors:** Saki Kasai, Masumi Nagata, Keisuke Jimbo, Nobuyasu Arai, Yuichiro Miyake, Mitsuyoshi Suzuki, Takahiro Kudo, Go Miyano, Hiromichi Shoji

**Affiliations:** ^1^ Department of Pediatrics Juntendo University Faculty of Medicine Tokyo Japan; ^2^ Department of Pediatric Surgery Juntendo University School of Medicine Tokyo Japan; ^3^ Department of Pediatrics and Neonatology Juntendo University Shizuoka Hospital Shizuoka Japan

**Keywords:** child, double‐balloon enteroscopy, Meckel diverticulum, radionuclide imaging, ultrasonography

## Abstract

Scintigraphy‐negative Meckel's diverticulum (MD) is diagnostically challenging in children, particularly when the underlying pathology differs between cases. Technetium‐99^m^ pertechnetate scintigraphy is widely used for diagnosis; however, false‐negative results may occur when ectopic gastric mucosa is absent or when the diverticulum is inverted. We report two pediatric cases with contrasting scintigraphy‐negative MD in which intestinal ultrasound (IUS) suggested target lesions, and double balloon enteroscopy (DBE) enabled direct diagnosis and preoperative localization.

Case 1 was a 13‐year‐old boy with recurrent abdominal pain and hematochezia. IUS revealed localized ileal wall thickening with a hyperechoic polypoid lesion, and transanal DBE identified a pedunculated ulcerated mass approximately 80 cm proximal to the ileocecal valve. Surgical pathology confirmed inverted MD with ectopic gastric mucosa. Case 2 was an 11‐year‐old girl with recurrent abdominal pain initially treated as ileitis. IUS revealed localized ileal wall thickening with increased vascularity and a blind‐ending tubular structure, and DBE showed a double‐lumen appearance consisting of the true ileal lumen and a blind‐ending diverticular lumen. Pathology confirmed MD without ectopic mucosa. DBE enabled direct diagnosis and preoperative marking without complications in both cases. These cases suggest that IUS may help identify target lesions and that DBE is useful for definitive diagnosis and preoperative localization in children with suspected MD despite negative scintigraphy.

**Abbreviations**
CT: computed tomography
DBE: double‐balloon enteroscopy
IUS: intestinal ultrasound
MD: Meckel's diverticulum.

## 1 Introduction

Meckel's diverticulum (MD) is a true diverticulum caused by incomplete obliteration of the vitelline duct and is the most common congenital gastrointestinal anomaly, occurring in approximately 2% of the population [1]. It arises from the antimesenteric border of the ileum and contains all layers of the intestinal wall. Although ectopic gastric mucosa is frequently associated with bleeding, its presence is not required for the pathological diagnosis of MD. Technetium‐99^m^ pertechnetate scintigraphy is the standard diagnostic modality for pediatric MD, with a pooled sensitivity of 80% and specificity of 95% in a meta‐analysis of 1115 pediatric cases [2]. However, false‐negative scintigraphy may occur when ectopic gastric mucosa is absent or when anatomical factors, such as diverticular inversion, obscure tracer accumulation [3]. Contrast‐enhanced computed tomography (CT) has limited sensitivity for detecting MD [4]. Double‐balloon enteroscopy (DBE) enables direct observation and preoperative marking, with a diagnostic yield exceeding 80% for MD [5, 6], and has been shown to be safe in pediatric patients [7]. Intestinal ultrasound (IUS) is a noninvasive, radiation‐free modality that may provide useful clues in children with suspected MD [8], although its role in scintigraphy‐negative cases remains unclear. Herein, we report two pediatric cases of scintigraphy‐negative MD with distinct pathological mechanisms. In both cases, IUS suggested localized ileal abnormalities, and DBE enabled direct identification and preoperative marking before surgical resection.

## 2 Case Report

### Case 1

**FIGURE 1:**
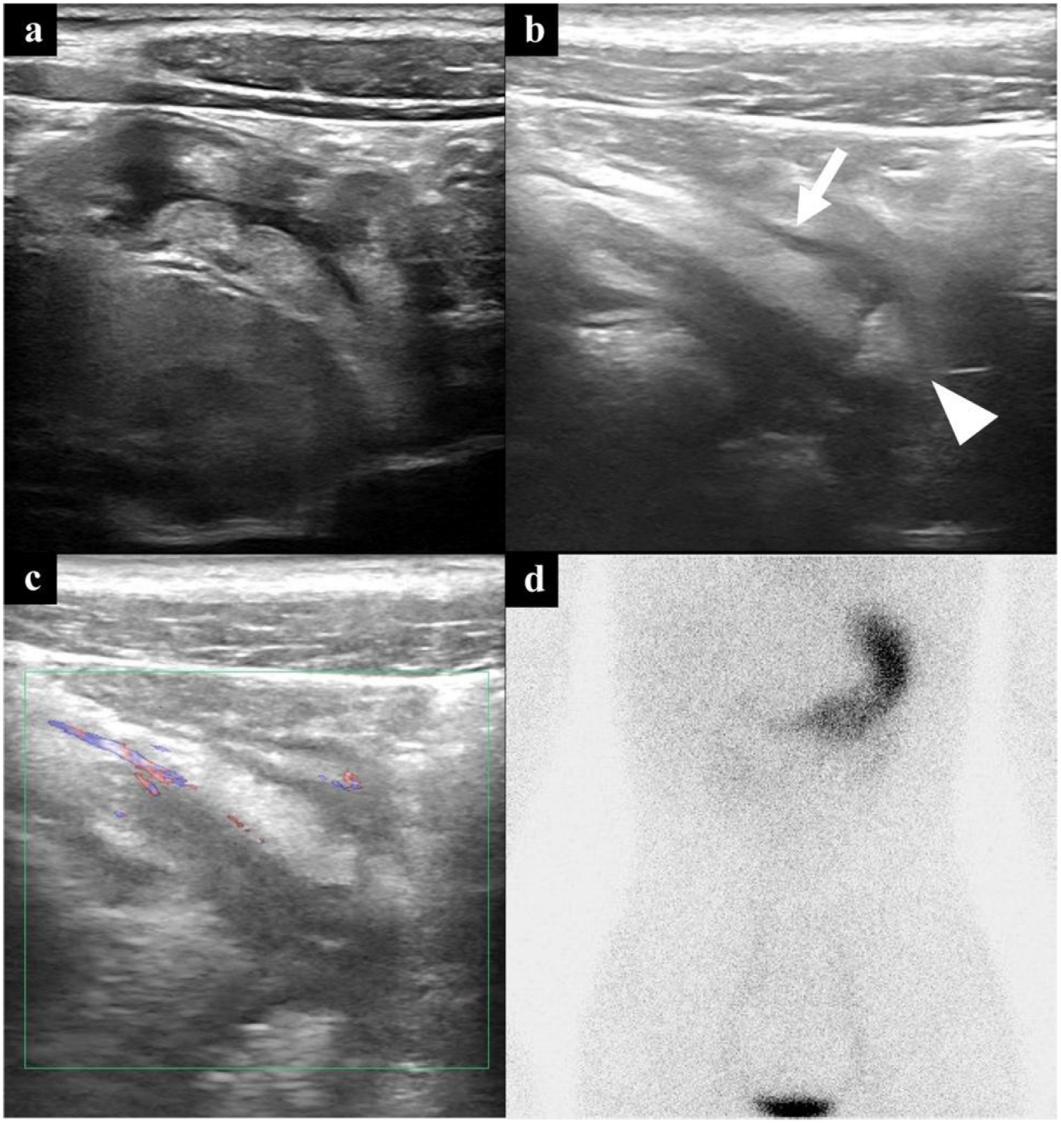
Ultrasonographic and Technetium‐99^m^ pertechnetate scintigraphy findings in Case 1. (a) Localized thickening of the ileal wall was observed. (b) A mass‐like structure with an intussusception‐like configuration (arrow) was identified, with a hyperechoic region at its tip (arrowhead) suggestive of an inverted diverticulum. (c) Advanced dynamic flow (a wide‐band Doppler imaging mode) demonstrated detectable intralesional blood flow, and the surrounding mesenteric fat showed increased echogenicity consistent with inflammation. (d) No definite abnormal uptake was detected on Technetium‐99^m^ pertechnetate scintigraphy.

**FIGURE 2:**
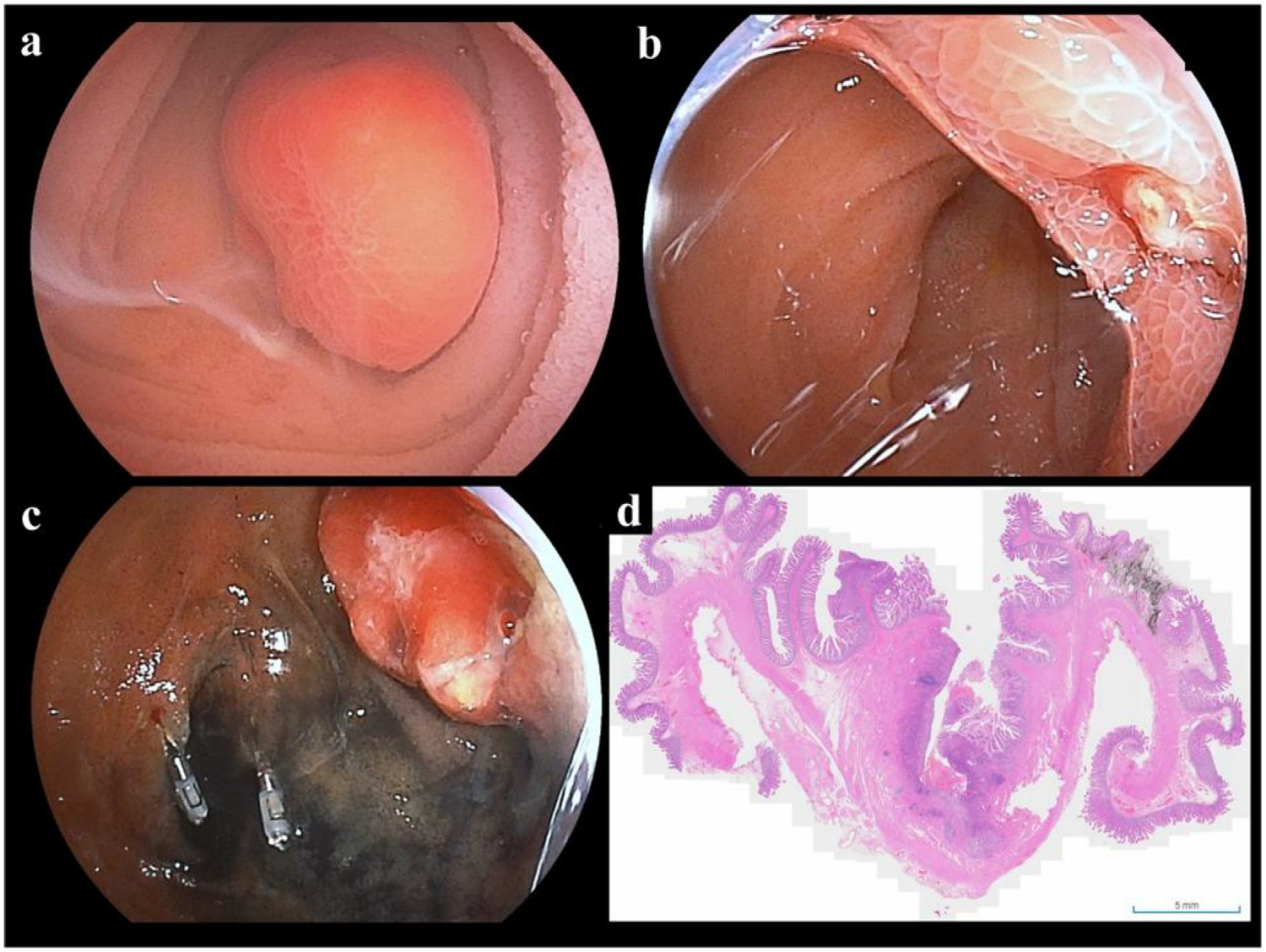
Transanal double‐balloon enteroscopy and histopathological findings in Case 1. (a) A pedunculated intraluminal mass was identified approximately 80 cm proximal to the ileocecal valve. (b) An ulcer was present at the tip of the pedunculated mass. (c) Tattooing and clipping were performed for localization. (d) Histopathological findings of the resected lesion (hematoxylin and eosin staining). The histology shows a true small‐intestinal diverticulum involving all layers of the intestinal wall (mucosa, submucosa, muscularis propria, and serosa) and containing ectopic gastric mucosa, consistent with a Meckel's diverticulum. Scale bar: 5 mm.

A 13‐year‐old boy had recurrent abdominal pain, vomiting, and hematochezia since the age of 12 years. Physical examination showed periumbilical tenderness. Previous colonoscopy and contrast‐enhanced CT revealed no abnormalities, and symptoms improved temporarily with conservative treatment. Eleven months after symptom onset, IUS with an 18‐MHz linear probe (Aplio i800; Canon Medical Systems, Tochigi, Japan) demonstrated localized ileal wall thickening (Figure 1a) and a polypoid lesion with a hyperechoic tip (Figure 1b,c), suggesting an ileal mass lesion. Scintigraphy showed no abnormal tracer accumulation (Figure 1d). Because IUS had already identified a localized ileal mass‐like lesion requiring direct evaluation and preoperative localization, capsule endoscopy was not performed. Transanal DBE under intravenous sedation with an EN‐580T enteroscope (Fujifilm, Tokyo, Japan) identified a pedunculated intraluminal mass with tip ulceration approximately 80 cm proximal to the ileocecal valve (Figure 2a–c). The ulcerated tip may have corresponded to the hyperechoic area on IUS. Tattooing and clip placement were performed for localization. Laparoscopy readily identified the tattooed segment, and histopathology confirmed MD with ectopic gastric mucosa (Figure 2d).

### Case 2

**FIGURE 3:**
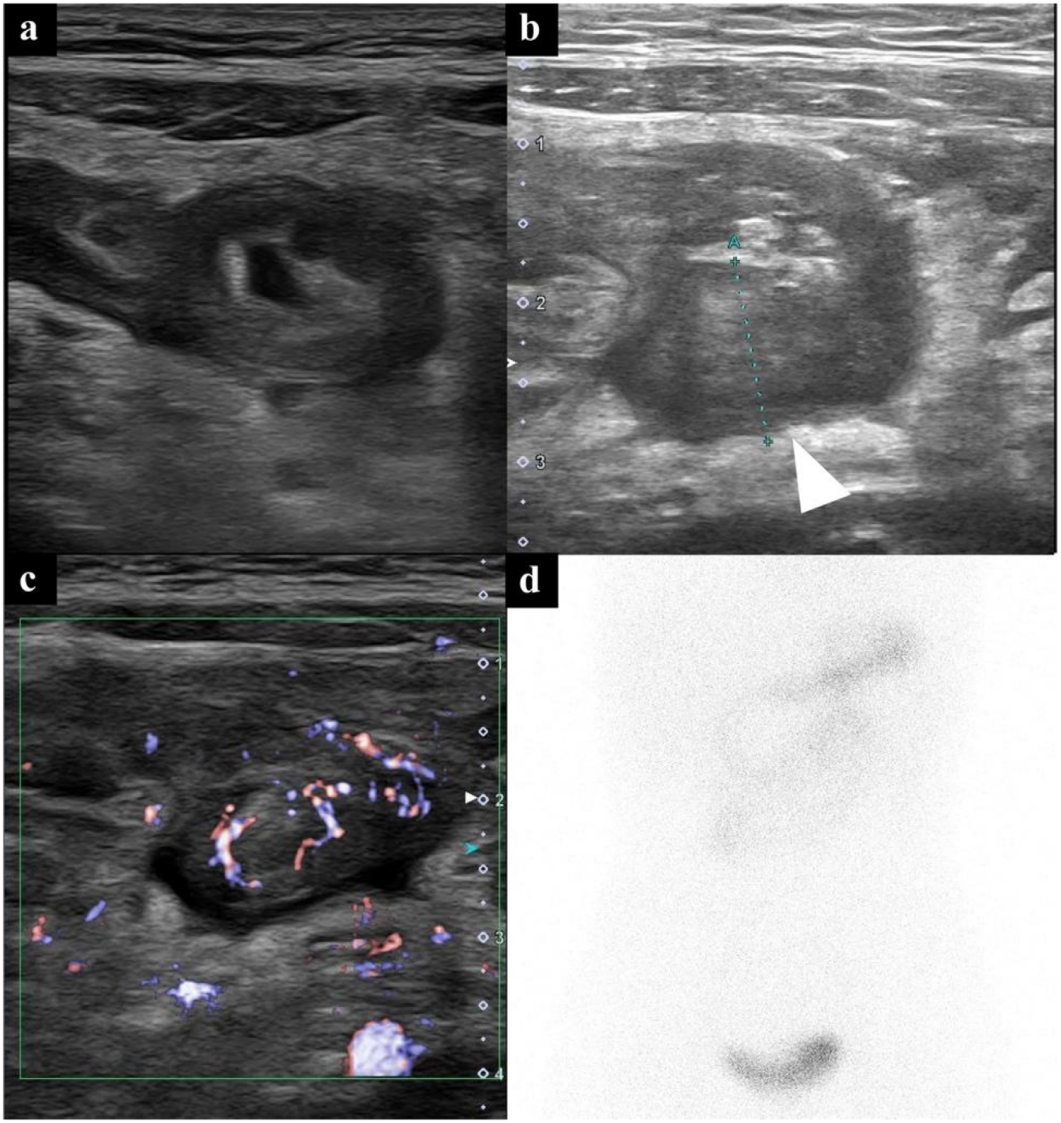
Ultrasonographic and Technetium‐99^m^ pertechnetate scintigraphy findings in Case 2. (a) Localized thickening of the ileal wall was observed. (b) A blind‐ending tubular structure (arrowhead) was visualized, consistent with a diverticular lesion. (c) Advanced dynamic flow demonstrated increased intralesional blood‐flow signals, and the surrounding mesenteric fat showed enhanced echogenicity consistent with inflammation. (d) No definite abnormal uptake was detected on Technetium‐99^m^ pertechnetate scintigraphy.

**FIGURE 4:**
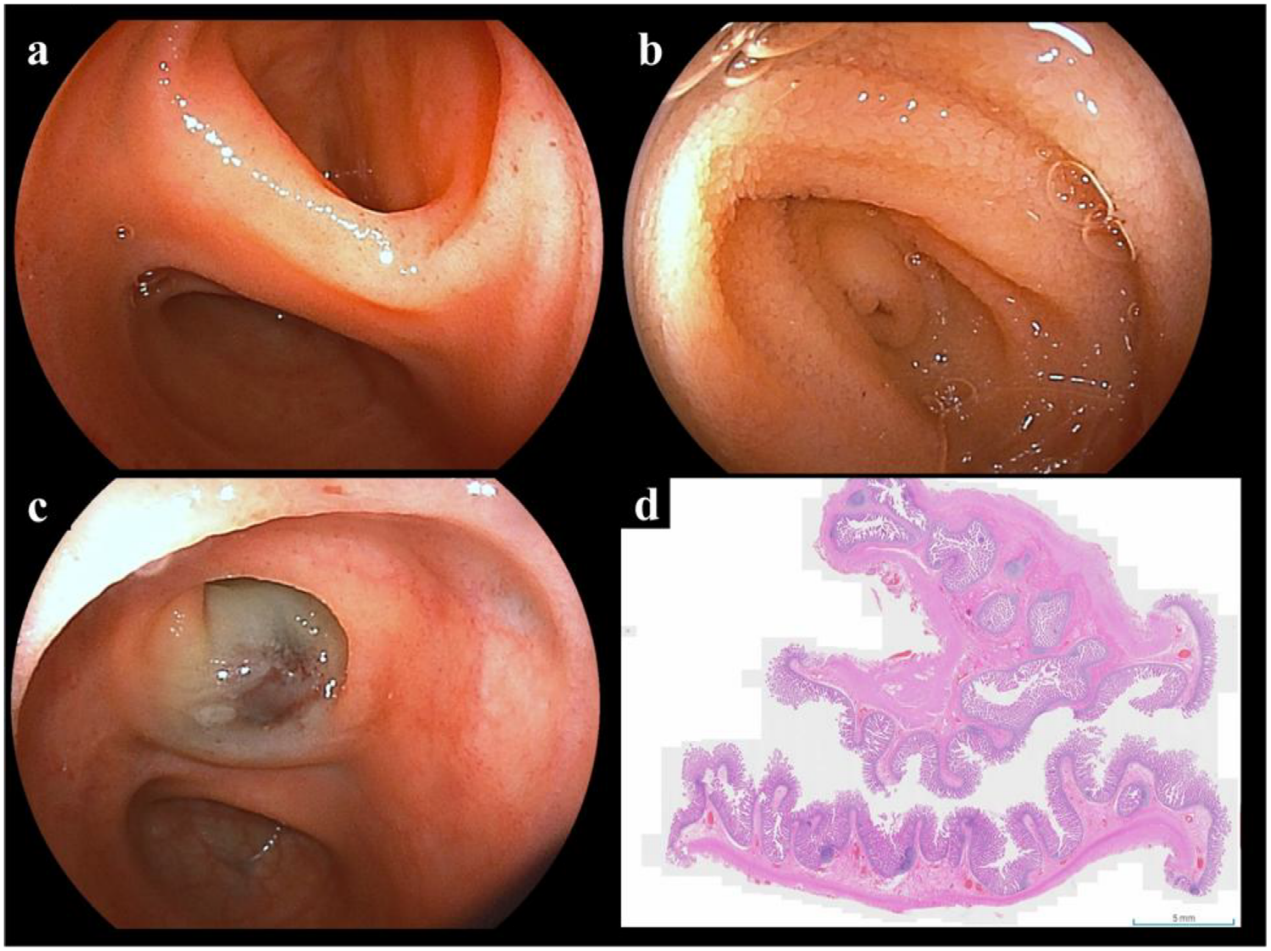
Transanal double‐balloon enteroscopy and histopathological findings in Case 2. (a) A double‐lumen appearance was visualized in the small intestine approximately 80 cm proximal to the ileocecal valve, with the true ileal lumen and a suspected diverticular lumen seen side by side. (b) The suspected diverticular lumen terminated blindly. (c) Tattooing was performed at the blind‐ending segment. (d) Histopathological findings of the resected lesion (hematoxylin and eosin staining). The histology shows a true small‐intestinal diverticulum involving all layers of the intestinal wall (mucosa, submucosa, muscularis propria, and serosa) without ectopic gastric mucosa, consistent with a Meckel's diverticulum. Scale bar: 5 mm.

An 11‐year‐old girl presented with recurrent abdominal pain and nausea. Physical examination showed lower abdominal tenderness. Initial CT showed mild terminal ileal wall thickening, and she was treated conservatively for ileitis with transient improvement. As Crohn's disease was initially considered owing to recurrent abdominal pain, mild growth impairment, and terminal ileal wall thickening, upper, lower, and capsule endoscopies were performed. These revealed no abnormalities, and retrospective review of the capsule images showed no definite findings suggestive of MD. Six months after the initial presentation, the symptoms persisted despite the negative endoscopic workup; therefore, IUS with an 11‐MHz linear probe (Aplio i800) was performed, focusing on the site of abdominal pain. It demonstrated persistent localized ileal wall thickening, a blind‐ending tubular structure (Figure 3a,b), and increased vascularity around the lesion (Figure 3c), raising suspicion of MD. Scintigraphy was negative (Figure 3d). Transanal DBE (EN‐580T) under intravenous sedation revealed a double‐lumen appearance approximately 80 cm proximal to the ileocecal valve: one lumen represented the true ileal lumen, whereas the other terminated blindly, consistent with a diverticular lumen, without ulceration or bleeding (Figure 4a–c). The lesion was tattooed and surgically resected. Pathology revealed MD without ectopic mucosa (Figure 4d). Neither patient had DBE‐related complications, and both had uneventful postoperative courses without recurrence.

## 3 Discussion

This report describes two pediatric cases of scintigraphy‐negative MD with distinct pathological backgrounds, in which IUS suggested target lesions and DBE enabled direct diagnosis and preoperative localization. Case 1 involved an inverted diverticulum with ectopic gastric mucosa, whereas Case 2 involved a non‐inverted diverticulum without ectopic mucosa. These cases show that a negative scintigraphy result does not exclude MD, particularly when symptoms persist or the clinical presentation is atypical.

The mechanisms of false‐negative scintigraphy differed between the two cases. In Case 1, inversion may have altered the anatomical configuration, luminal exposure, or local tracer retention despite the presence of ectopic gastric mucosa [3]. In Case 2, the absence of ectopic gastric mucosa directly explained the negative scan. Thus, scintigraphy‐negative MD is not a uniform diagnostic entity but may arise from distinct pathological mechanisms.

DBE was important for diagnosis and surgical planning in these cases, but it was not used as a first‐line modality. Rather, it was selected when symptoms persisted or recurred despite negative or inconclusive conventional examinations and when direct visualization or preoperative localization was expected to affect management. In Case 1, IUS had demonstrated a localized ileal mass‐like lesion requiring direct assessment and marking. In Case 2, symptoms persisted despite negative capsule endoscopy and scintigraphy, and IUS suggested a localized small‐bowel lesion. DBE identified a pedunculated ulcerated mass in Case 1 and a double‐lumen appearance, consisting of the true ileal lumen and a blind‐ending diverticular lumen, in Case 2. Preoperative marking enabled reliable laparoscopic localization in both patients. No DBE‐related complications occurred, consistent with previous pediatric safety data. In a large study of 257 pediatric procedures, the overall complication rate was 1.9% when double‐balloon endoscopic retrograde cholangioscopy was excluded [7]. Previous studies have also shown the diagnostic value of DBE for MD, including scintigraphy‐negative cases [9, 10].

IUS detected localized distal small‐bowel abnormalities in both patients despite negative scintigraphy and unrevealing or nonspecific CT findings. The examinations were performed by pediatric gastroenterologists experienced in intestinal ultrasonography. Lesion localization was initially suggested by targeted IUS over the symptomatic right lower abdominal area, together with sonographic features compatible with distal small‐bowel involvement, including relatively inconspicuous Kerckring folds, and was subsequently confirmed by transanal DBE approximately 80 cm proximal to the ileocecal valve in both cases. Although IUS cannot definitively distinguish jejunum from ileum and is operator‐dependent, it helped identify localized distal small‐bowel abnormalities and guide targeted DBE. General findings such as focal wall thickening, vascularity, and hyperechoic mesenteric fat may be reproducible in centers familiar with pediatric bowel ultrasound, whereas subtle findings such as a hyperechoic polypoid tip or blind‐ending tubular structure may require expertise.

The discrepancy between capsule endoscopy and IUS in Case 2 may reflect their complementary roles. Capsule endoscopy was initially performed because Crohn's disease was suspected, but no abnormality was observed even on retrospective review. Capsule endoscopy provides passive, non‐steerable mucosal imaging and may miss a non‐inverted MD without ulceration or bleeding if the diverticular orifice is not adequately visualized. In contrast, IUS can assess mural and extraluminal features, including focal wall thickening, a blind‐ending structure, vascularity, and surrounding inflammation.

Based on these cases, we propose a simplified workflow (Figure S1): scintigraphy is generally considered early in the diagnostic workup when MD is suspected. If scintigraphy is negative but symptoms persist, contrast‐enhanced CT and/or capsule endoscopy may be used according to the presentation. IUS is a noninvasive, radiation‐free modality that can be performed and repeated at any stage of the workup and may help identify a localized small‐bowel lesion that can serve as a target for DBE. When IUS suggests such a lesion, or when suspicion remains high despite negative noninvasive examinations, DBE may be considered for direct visualization and preoperative marking.

In conclusion, scintigraphy‐negative MD may result from distinct pathological mechanisms, including diverticular inversion despite ectopic gastric mucosa and absence of ectopic gastric mucosa. IUS may serve as a noninvasive adjunct for identifying target lesions, whereas DBE can provide definitive diagnosis and preoperative localization in children with persistent suspicion of MD despite negative scintigraphy.

## Author Contributions

**Saki Kasai** and **Masumi Nagata** contributed to writing the original draft, reviewing and editing, data curation, and investigation. **Masumi Nagata** also contributed to conceptualization, supervision, and project administration. **Keisuke Jimbo** contributed to the investigation, resources, and project administration. **Nobuyasu Arai**, **Yuichiro Miyake**, and **Mitsuyoshi Suzuki** contributed to the investigation and resources. **Takahiro Kudo**, **Go Miyano**, and **Hiromichi Shoji** contributed to conceptualization and supervision. All authors have read and agreed to the final version of the manuscript.

## Funding

The authors have nothing to report.

## Ethics Statement

The authors have nothing to report.

## Consent

Written consent for publication was obtained from the patients’ parents.

## Conflicts of Interest

The authors declare no conflicts of interest.

## Supporting information




**FIGURE S1**: Proposed diagnostic workflow for suspected pediatric Meckel's diverticulum, with emphasis on scintigraphy‐negative cases. When Meckel's diverticulum (MD) is clinically suspected, Technetium‐99^m^ pertechnetate scintigraphy is generally considered early in the diagnostic workup. If scintigraphy is positive, the patient proceeds to surgical management. If scintigraphy is negative but symptoms persist, further evaluation with contrast‐enhanced CT and/or capsule endoscopy may be performed according to the clinical presentation. Intestinal ultrasound (IUS), a noninvasive and radiation‐free modality, can be performed and repeated at any stage of the workup and may help identify a localized small‐bowel lesion that can serve as a target for subsequent evaluation. When IUS suggests such a lesion, or when clinical suspicion remains high despite negative noninvasive examinations, double‐balloon enteroscopy (DBE) may be considered for direct visualization and preoperative marking, followed by surgical resection. DBE is not used as a first‐line modality, but is selected when symptoms persist or recur despite negative or inconclusive conventional examinations and when direct visualization or preoperative localization is expected to affect management.
