# Supplementary material for: Two Contrasting Scintigraphy‐Negative Pediatric Meckel's Diverticula Identified and Localized by Intestinal Ultrasound and Double‐Balloon Enteroscopy: A Report of Two Cases
Source: DEN Open. 2026 Jul 13;7(1):e70379. doi: 10.1002/deo2.70379 (PMC13359145; doi:10.1002/deo2.70379)
Supplement: Supplementary file 1 — FIGURE S1: Proposed diagnostic workflow for suspected pediatric Meckel's diverticulum, with emphasis on scintigraphy‐negative cases. When Meckel's diverticulum (MD) is clinically suspected, Technetium‐99m pertechnetate scintigraphy is generally considered early in the diagnostic workup. If scintigraphy is positive, the patient proceeds to surgical management. If scintigraphy is negative but symptoms persist, further evaluation with contrast‐enhanced CT and/or capsule endoscopy may be performed according to the clinical presentation. Intestinal ultrasound (IUS), a noninvasive and radiation‐free modality, can be performed and repeated at any stage of the workup and may help identify a localized small‐bowel lesion that can serve as a target for subsequent evaluation. When IUS suggests such a lesion, or when clinical suspicion remains high despite negative noninvasive examinations, double‐balloon enteroscopy (DBE) may be considered for direct visualization and preoperative marking, followed by surgical resection. DBE is not used as a first‐line modality, but is selected when symptoms persist or recur despite negative or inconclusive conventional examinations and when direct visualization or preoperative localization is expected to affect management. [file DEO2-7-e70379-s001.docx]

Supplementary Figure S1. Proposed diagnostic workflow for suspected pediatric Meckel’s diverticulum, with emphasis on scintigraphy-negative cases
